# A Comprehensive miRNome Analysis of Macrophages Isolated from db/db Mice and Selected miRNAs Involved in Metabolic Syndrome-Associated Cardiac Remodeling

**DOI:** 10.3390/ijms22042197

**Published:** 2021-02-23

**Authors:** Justyna Niderla-Bielińska, Aneta Ścieżyńska, Aneta Moskalik, Ewa Jankowska-Steifer, Krzysztof Bartkowiak, Mateusz Bartkowiak, Ewelina Kiernozek, Anna Podgórska, Bogdan Ciszek, Barbara Majchrzak, Anna Ratajska

**Affiliations:** 1 Department of Histology and Embryology, Collegium Anatomicum, Medical University of Warsaw, 02-004 Warsaw, Poland; jniderla@wum.edu.pl (J.N.-B.); asciezynska@wum.edu.pl (A.Ś.); ewa.jankowska-steifer@wum.edu.pl (E.J.-S.); 2 Postgraduate School of Molecular Medicine, Collegium Anatomicum, Medical University of Warsaw, 02-004 Warsaw, Poland; aneta.moskalik@gmail.com; 3 Student Scientific Group, Department of Histology and Embryology, Collegium Anatomicum, Medical University of Warsaw, 02-004 Warsaw, Poland; krzysztof_32@onet.eu (K.B.); mateusz.bartkowiak@wum.edu.pl (M.B.); 4 Department of History of Medicine, Medical University of Warsaw, 00-575 Warsaw, Poland; 5 Department of Immunology, Faculty of Biology, University of Warsaw, 02-096 Warsaw, Poland; ekiernozek@biol.uw.edu.pl; 6 Molecular Biology Laboratory, Department of Medical Biology, Cardinal Stefan Wyszyński Institute of Cardiology, 04-628 Warsaw, Poland; apodgorska@ikard.pl; 7 Department of Clinical Anatomy, Collegium Anatomicum, Medical University of Warsaw, 02-004 Warsaw, Poland; bogdan.ciszek@wum.edu.pl; 8 Department of Pathology, Collegium Anatomicum, Medical University of Warsaw, 02-004 Warsaw, Poland; bmajchrzak@wum.edu.pl

**Keywords:** cardiac macrophages, miRNA, metabolic syndrome, myocardial remodeling

## Abstract

Cardiac macrophages are known from various activities, therefore we presume that microRNAs (miRNAs) produced or released by macrophages in cardiac tissue have impact on myocardial remodeling in individuals with metabolic syndrome (MetS). We aim to assess the cardiac macrophage miRNA profile by selecting those miRNA molecules that potentially exhibit regulatory functions in MetS-related cardiac remodeling. Cardiac tissue macrophages from control and db/db mice (an animal model of MetS) were counted and sorted with flow cytometry, which yielded two populations: CD45^+^CD11b^+^CD64^+^Ly6C^hi^ and CD45^+^CD11b^+^CD64^+^Ly6C^low^. Total RNA was then isolated, and miRNA expression profiles were evaluated with Next Generation Sequencing. We successfully sequenced 1400 miRNAs in both macrophage populations: CD45^+^CD11b^+^CD64^+^Ly6C^hi^ and CD45^+^CD11b^+^CD64^+^Ly6C^low^. Among the 1400 miRNAs, about 150 showed different expression levels in control and db/db mice and between these two subpopulations. At least 15 miRNAs are possibly associated with MetS pathology in cardiac tissue due to direct or indirect regulation of the expression of miRNAs for proteins involved in angiogenesis, fibrosis, or inflammation. In this paper, for the first time we describe the miRNA transcription profile in two distinct macrophage populations in MetS-affected cardiac tissue. Although the results are preliminary, the presented data provide a foundation for further studies on intercellular cross-talk/molecular mechanism(s) involved in the regulation of MetS-related cardiac remodeling.

## 1. Introduction

The diagnostic criteria for metabolic syndrome (MetS) have been modified since 1998, when they were first established by the WHO [1]. According to the latest data, MetS is defined as a set of metabolic abnormalities that include at least three of the following risk factors: insulin resistance, type 2 diabetes (T2D), obesity, elevated triglycerides, hypertension and hyperglycemia [2]. The prevalence of MetS is reported to be approximately 30% within the human population. Metabolic abnormalities, especially obesity, hyperglycemia, and hyperlipidemia consequently lead to the development of T2D, atherosclerosis, and finally cardiovascular disease (CVD) leading to heart failure (HF). Abnormal myocardial remodeling in MetS-affected individuals involves microvascular dysfunction, followed by impaired blood flow, prolonged chronic inflammation, tissue fibrosis, increased myocardial wall stiffness causing diastolic dysfunction, repeated episodes of vasoconstriction, and reperfusion injury [3,4,5]. The current knowledge on the molecular, structural, and cellular interaction in myocardial tissue of MetS individuals and on specific treatments for patients suffering from HF and MetS is still incomplete. Various MetS-mimicking animal models, including db/db mice, facilitate research in this field. Recently, cardiac tissue macrophages have been reported to play diverse roles in maintaining cardiac tissue homeostasis in steady-state [6,7] and disease [8,9,10], and may contribute to CVD progression by their involvement in diastolic myocardial dysfunction [6,11]. Several macrophage subpopulations selected on the basis of their cell surface markers, including F4/80^+^, CD11b^+^, CD64^+^, MerTK^+^, and CCR2^+^ are involved in the regulation of fibrosis, hypertrophy, inflammation, angiogenesis, lymphangiogenesis, endothelial cell regeneration, respiratory burst, in addition to classical function in the innate and adaptive immune responses [12]. Recently, single-cell transcriptomic analysis was used for macrophage population selection and revealed the existence of various phenotypic subsets among macrophages in a healthy myocardium. Moreover, genetic profile analyses would help select other cardiac macrophage populations and illustrate the potentially beneficial or detrimental effects of macrophages on heart function in CVD [13]. We therefore aimed to test the hypothesis that cardiac macrophages might promote adverse myocardial remodeling in MetS.

MicroRNAs (miRNAs) are small, non-coding RNAs, which negatively regulate gene expression by translational inhibition or mRNA decay. The miRNA sequence is often not perfectly complementary to the target sequence and thus a single miRNA can affect the expression of multiple mRNAs. Additionally miRNAs can be secreted to the extracellular space encapsulated in extracellular vesicles and can be taken up by neighboring or distant cells [14]. Recently, numerous miRNAs were described as involved in the pathogenesis of HF in MetS. In vitro and in vivo studies state that abnormal expression of miRNAs may cause endothelial cell dysfunction and impair angiogenesis. Some miRNAs can also be diagnostic biomarkers of MetS and microvascular complications, as they are secreted from cells, and their concentration in the serum is altered [15]. Additionally, miRNAs can be potential targets for MetS and HF therapy [16,17].

The possible genetic targets and functions of the miRNAs expressed by cardiac macrophages in MetS are still unexplored. In the present study, we investigated miRNA expression profiles in two distinct populations of macrophages isolated from the cardiac tissue of db/db mice via Next Generation Sequencing. Db/db mice are an animal model of MetS, established by a genetic mutation of the leptin receptor, and presenting obesity, insulin resistance, and T2D [18]. The goal of this study was to identify changes in miRNA expression in myocardial macrophages from MetS and healthy control mice and to determine miRNAs of interest for further studies. Our results show that the expression of numerous miRNAs involved in angiogenesis, fibrosis, and inflammation is altered in the MetS heart, shedding a new light on possible molecular implications in cardiac remodeling in MetS patients and opening new pathways for further studies in this field.

## 2. Results

### 2.1. Db/db Mice Exhibit Obesity and Hyperglycemia

**Figure 1:**
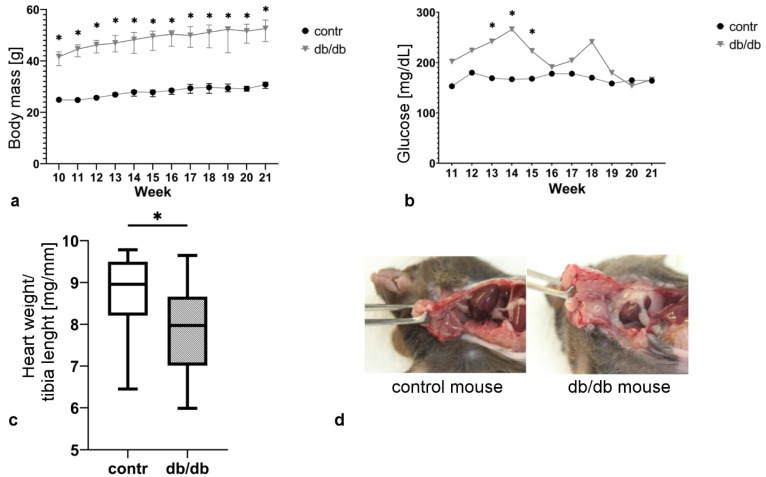
The development of MetS symptoms in db/db mice. (**a**) body weight gain; (**b**) blood glucose level; (**c**) normalized heart weight; (**d**) pericardial adipose tissue in control and db/db mice. Values are mean ± S.E. (*n* = 6); *, *p* < 0.05 versus control group as determined by Wilcoxon signed-rank test.

The body weight of both control and db/db mice rose gradually, with db/db mouse body weight significantly higher and reaching about 50 g in week 21. Body weight exhibited a higher variability in db/db mice compared with that in control animals (Figure 1a). Blood glucose levels were also increased in db/db mice, although the difference was not always statistically significant due to high inter-individual diversity of results in the db/db group (Figure 1b). On the other hand, heart weight values in week 21 were significantly lower in the db/db group, when normalized for tibia length (Figure 1c). We also observed more abundant pericardial and abdominal adipose tissue in db/db mice compared with those parameters in control mice (Figure 1d).

### 2.2. The Significant Decrease in Macrophage Numbers in db/db vs. Control Mouse Hearts

**Figure 2:**
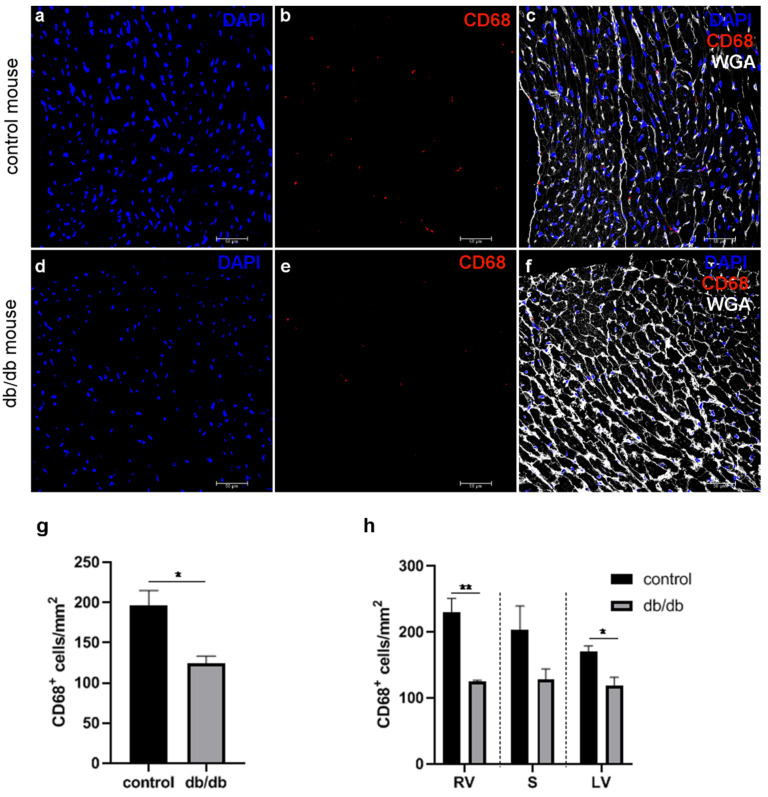
Confocal microscope analysis of macrophage density in the LV of control (**a**–**c**) and db/db (**d**–**f**) mice. Macrophages were visualized with anti-CD68 antibody. WGA (wheat germ agglutinin) was used to mark microvessels and cardiac myocyte boundaries. Figures were chosen among at least three independent stainings; the number of CD68 positive macrophages per mm^2^ in whole (**g**) and in specific areas of heart (**h**) was calculated. Data are expressed as mean number of cells per area (mm^2^), calculated within three independent stainings and 10 randomly selected regions of interest per staining; RV—right ventricle, LV—left ventricle, S—interventricular septum. * *p* < 0.05, ** *p* < 0.01.

**Figure 3:**
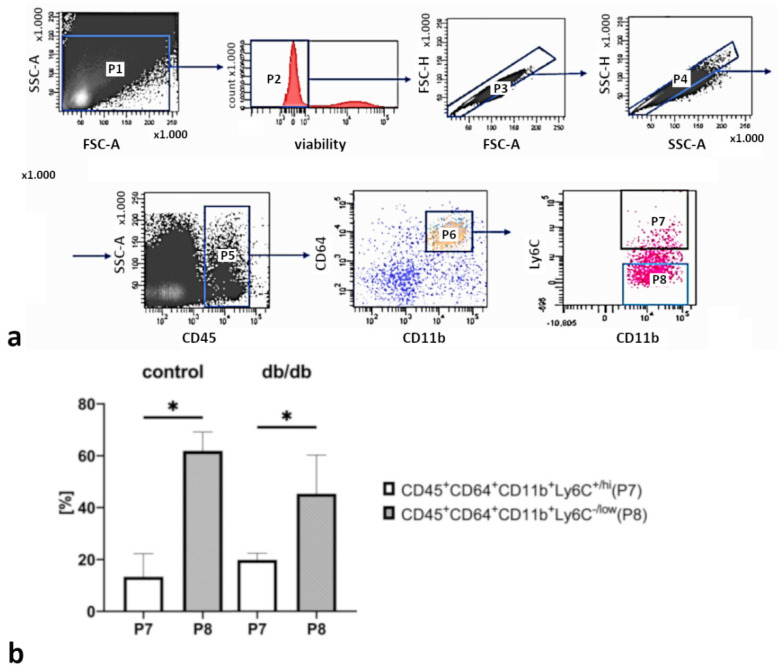
Sorting strategy (**a**) and macrophage subpopulations count in db/db and control mice (**b**). Gates: P1—whole cell count after enzymatic digestion of cardiac tissue; P2—viable cells; P3 and P4—doublet exclusion; P5—CD45^+^; P6—CD45^+^CD11b^+^CD64^+^. Finally, two populations were gated: P7—CD45^+^CD63^+^CD11b^+^Ly6C^+/hi^ and P8—CD45^+^CD63^+^CD11b^+^Ly6C^+/low^. Values are mean ± S.E. (*n* = 6); *, *p* < 0.05 versus control group as determined by Wilcoxon signed-rank test.

Evaluation of CD68-positive macrophage locations on myocardial cryosections from healthy and db/db mice in a confocal microscope revealed that these cells were evenly distributed within the myocardial wall (Figure 2a–f), although the number of macrophages was lower in db/db group when whole hearts were analyzed (Figure 2g). Detailed analysis of specified areas of db/db mouse hearts showed a statistically significant reduction in the macrophage number in the left and right ventricular walls compared with the macrophage number in these areas of control myocardia. The density of CD68-positive cells in the interventricular septum was lower in db/db mice compared with that in control mice, but the difference was not statistically significant (Figure 2h). Flow cytometry analysis confirmed these observations (Figure 3a,b). Two macrophage populations were separated in control and db/db mice: CD45^+^CD11b^+^CD64^+^Ly6C^hi^ and CD45^+^CD11b^+^CD64^+^Ly6C^low^. The CD45^+^CD11b^+^CD64^+^Ly6C^hi^ population contained a lower number of macrophages than the CD45^+^CD11b^+^CD64^+^Ly6C^low^ population both in control and db/db mice (Figure 3b).

### 2.3. miRNA Expression Profile Changes in db/db Mouse Cardiac Macrophages but Not in Cardiac Tissue

**Figure 4:**
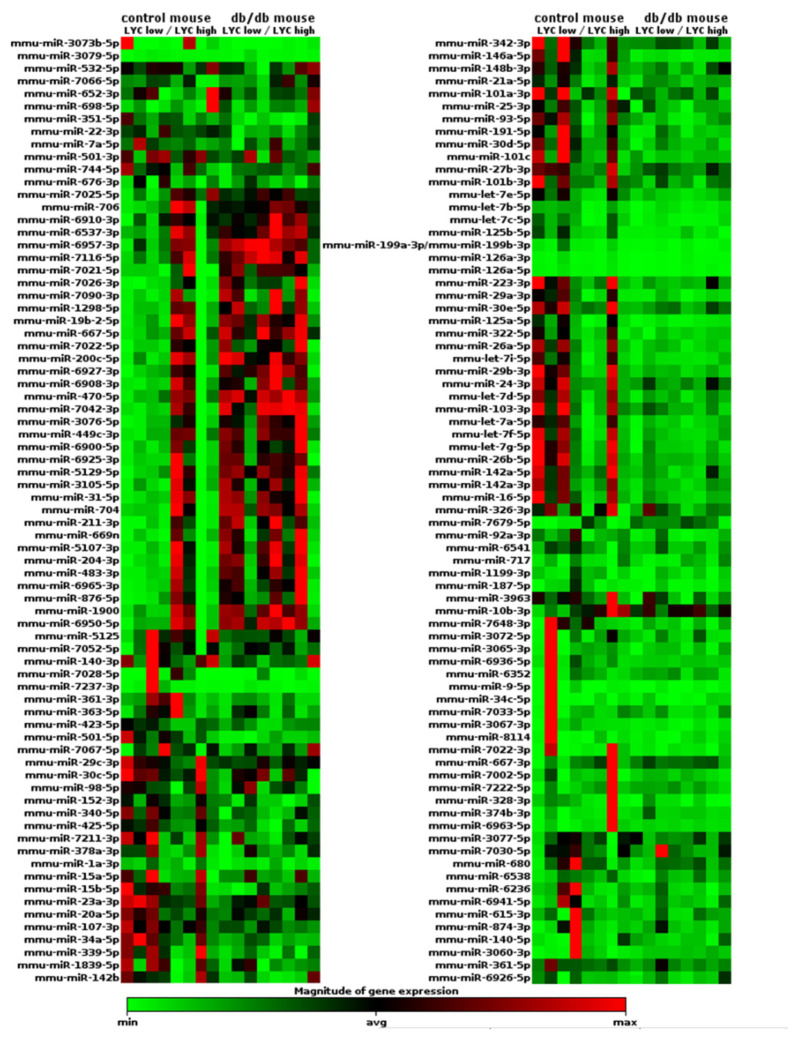
Heat map of miRNA expression data obtained with NGS from two populations of cardiac macrophages isolated from control (*n* = 4) and db/db (*n* = 4) mouse hearts. Results were normalized with the “Trimmed Mean of M” method and heat map was prepared on the basis of geNorm method. Relative miRNA expression is depicted according to the color scale shown below. Red indicates upregulation; green, downregulation.

**Figure 5:**
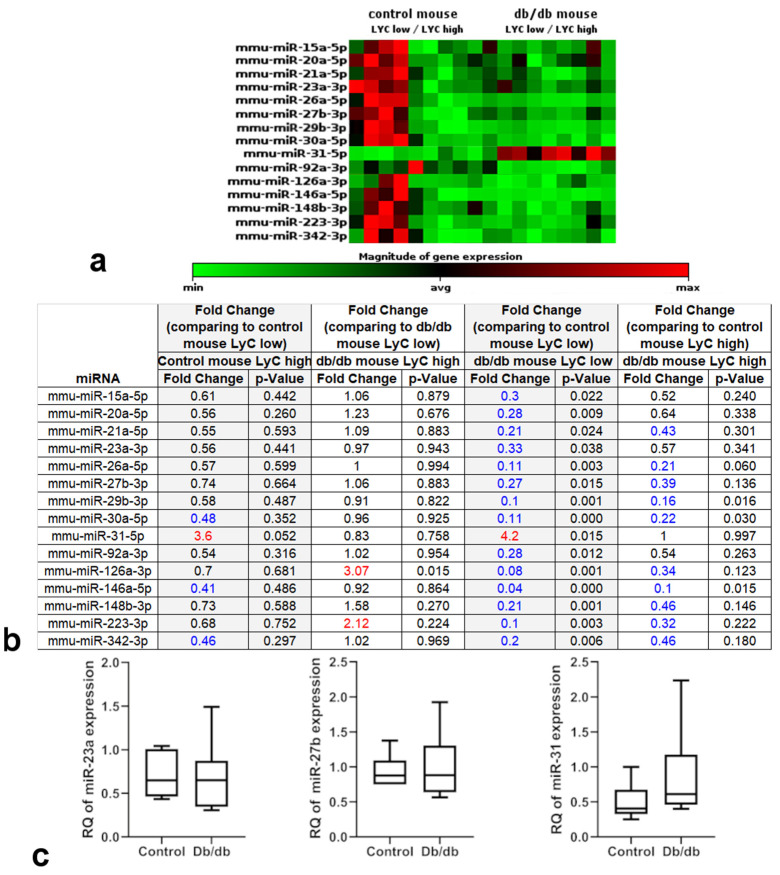
Heat map of selected miRNA expression data (**a**) obtained with NGS from two populations of cardiac macrophages isolated from control (*n* = 4) and db/db (*n* = 4) mouse hearts. Results were normalized with the “Trimmed Mean of M” method and heat map was prepared based on the geNorm method. Relative miRNA expression is depicted according to the color scale shown below. Red indicates upregulation; green, downregulation. Table (**b**) shows selected miRNAs and fold change, which is the normalized miRNA expression in each Test Sample divided by the normalized miRNA expression in the Control Sample. Numbers in blue indicate downregulation, whereas in red – upregulation of miRNA expression. *p*-values were calculated with a Bioconductor software package. (**c**) Selected miRNAs expression in cardiac tissue from db/db (*n* = 6) and control (*n* = 6) mice measured with relative quantitation (RQ) using a comparative C_T_ assay. Cardiac tissue from control mice was used as a calibrator. *p*-values were calculated with SAS 9.4 software.

We successfully sequenced 1400 miRNAs in both evaluated macrophage populations: CD45^+^CD11b^+^CD64^+^Ly6C^hi^ and CD45^+^CD11b^+^CD64^+^Ly6C^low^ (Supplementary Table S1). Out of the analyzed 1400 miRNAs, about 150 miRNAs significantly differed in db/db mice from those in control animals and also some of them were differentially expressed among two distinct macrophage populations—CD45^+^CD11b^+^CD64^+^Ly6C^hi^ and CD45^+^CD11b^+^CD64^+^Ly6C^low^. Interestingly differences between CD45^+^CD11b^+^CD64^+^Ly6C^hi^ and CD45^+^CD11b^+^CD64^+^Ly6C^low^ were more prominent in control mouse macrophages than in db/db animals (Figure 4). A manual search through the PubMed database showed at least 15 of these miRNAs to be possibly associated with MetS pathogenesis in cardiac tissue due to direct or indirect regulation of expression of mRNAs for proteins involved in angiogenesis (VEGF-A, Tie-2, AKT-3, SEMA6A, Sprouty2, IGF-1, KLF2, KLF4, endoglin, angiopoietin-2), fibrosis/extracellular matrix deposition (TGFβR2, SMAD4, Wnt, elastin, collagen, fibronectin, Snail1), inflammation (PPARα, IL-1, IL-6, TNFα, VCAM-1, NFκB), or lymphangiogenesis (Prox-1) (Table 1). Additionally, selected miRNAs can also affect macrophage function due to their influence on macrophage phenotype. Selected miRNAs, according to the literature data, may downregulate the expression of mRNA for proteins involved in anti-inflammatory (TNIP2, TNFAIP3, PTEN, KLF4, Cab39, IL10) or pro-inflammatory (JAK2, PDC4, CTFG, MIP-1β, SOCS3, ATF3, ATP1B1, ATP9A, RAIl4, Notch1, Nox2, Nlrp3, Pknox, Rasal, Nfat5 and Chi3l1) pathways, as well as angiogenesis (HIF-2α), phagocytosis (DNMTs) or lipid uptake (Chi3l1) (Table 2). We compared fold change in expression of selected miRNAs between CD45^+^CD11b^+^CD64^+^Ly6C^hi^ and CD45^+^CD11b^+^CD64^+^Ly6C^low^ macrophage populations, for both control and db/db mouse. There were no significant differences in miRNA levels between CD45^+^CD11b^+^CD64^+^Ly6C^hi^ and CD45^+^CD11b^+^CD64^+^Ly6C^low^ population in control mouse, except for miR-31-5p, which was upregulated in CD45^+^CD11b^+^CD64^+^Ly6C^hi^ population (although the difference was not statistically significant), and in db/db mouse except for miR-126a-3p which was also upregulated in CD45^+^CD11b^+^CD64^+^Ly6C^hi^ macrophages. All selected miRNAs, except for miR-31, were downregulated in db/db mouse cardiac tissue-derived CD45^+^CD11b^+^CD64^+^Ly6C^low^ macrophages compared with the miRNA levels in CD45^+^CD11b^+^CD64^+^Ly6C^low^ macrophage populations isolated from control hearts. All the differences were statistically significant. Interestingly, we did not observe significant differences in miRNA expression levels between CD45^+^CD11b^+^CD64^+^Ly6C^hi^ macrophages isolated from control hearts versus the same population from db/db mouse cardiac tissue, except for miR-30a-5p and miR146a-5p, which were downregulated (Figure 5a,b). Levels of selected miRNA expression (miR31, miR23 and miR27) in whole cardiac tissue were not affected (Figure 5c).

### 2.4. Morphological Analysis of Tissue Sections and Cell Suspensions Obtained from Control and db/db Mouse Hearts Showed Evidence of Cardiac Fibrosis, Inflammation, and Microvascular Rarefaction

**Figure 6:**
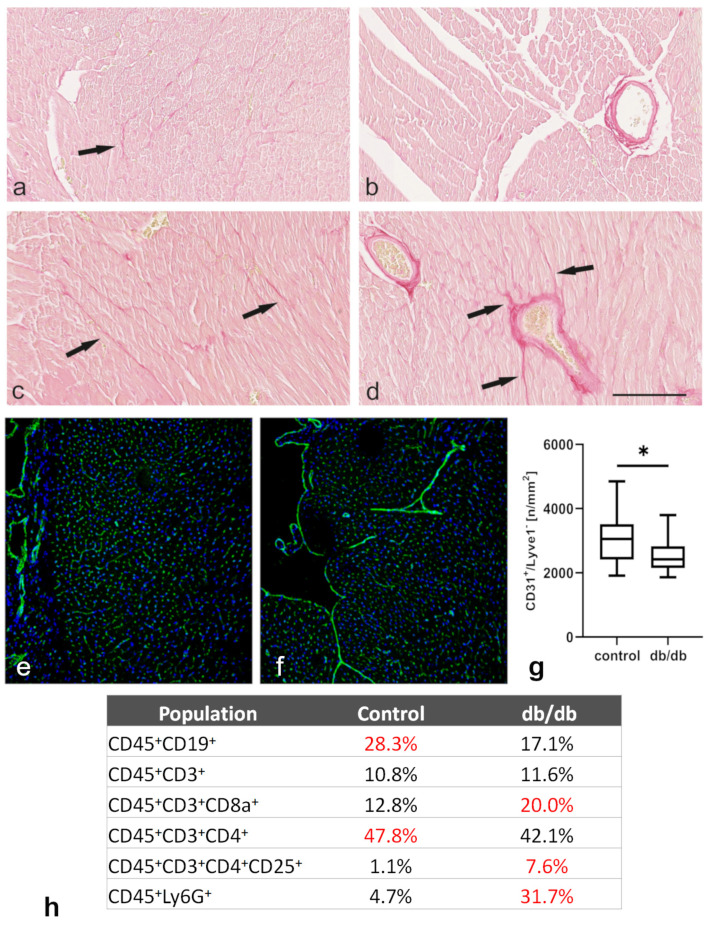
(**a**–**d**) Paraffin sections of control and db/db mouse hearts stained with Picrosirius red demonstrate collagen deposits. (**a**,**b**)—representative sections from control hearts with a slight interstitial and perivascular fibrosis stained in dark red; (**c**,**d**)—representative sections from db/db mice demonstrating a slight increase in interstitial and perivascular fibrosis, marked with arrows; scale bar—100 μm. (**e**–**g**) Density of CD31^+^Lyve-1^−^ cells in the left ventricular wall of control and db/db mice. (**e**,**f**) selected cryosections of control and db/db mouse hearts stained with anti-CD31 (green) and anti-Lyve-1 (red) antibodies, analyzed under a confocal microscope. (**g**) CD31^+^Lyve-1^−^ cell count per 1 mm^2^ in the left ventricular wall of control and db/db mouse hearts; * *p* < 0.05 versus control group as determined by Wilcoxon signed-rank test. (**h**) Flow cytometry analysis of leukocyte subpopulations in cell suspensions obtained from control and db/db mouse cardiac tissue. Table shows percentages of leukocyte subpopulations in total leukocyte population.

Histological sections of control hearts stained with Picrosirius red revealed a weak interstitial fibrosis and scant perivascular collagen deposits in the adventitia of medium-sized coronary vessels (Figure 6a,b). In db/db mice there was a slight increase in interstitial collagen deposits and perivascular fibrosis around vessels of the same diameter compared with the cardiac tissue in control animals (Figure 6c,d, marked with arrows). Light microscopy showed collagen deposits, which were stained dark red, forming branches towards interstitial cardiomyocytes positioned adjacent to coronary vessels (black arrows in d).

Confocal microscopic analysis of cardiac cryosections stained with anti-CD31 and anti-Lyve-1^−^ antibodies showed a significant decrease in CD31^+^Lyve-1^−^ microvessels in the left ventricles of db/db mice when compared with the microvessel number in control animals (Figure 6e–g). Moreover, the inflammatory cell profile assessed by flow cytometry analysis in cell suspensions obtained from cardiac tissue showed elevated numbers of monocytes, neutrophils, and granulocytes (CD45^+^Ly6g^+^) in db/db mice versus control animals. Interestingly, levels of T lymphocyte subpopulations were also elevated in db/db mouse cardiac tissue. Both cytotoxic T lymphocyte (CD45^+^CD3^+^CD8^+^) and regulatory T lymphocyte (CD45^+^CD3^+^CD8^+^, CD25^+^) counts were higher than in control mice, although the total number of T lymphocytes (CD45^+^CD3^+^) was unaffected. Finally, the lymphocyte B (CD45^+^CD19^+^) count was reduced in db/db mouse cardiac tissue when compared with that in control animals (Figure 6h).

## 3. Discussion

Db/db mice are widely used as an animal model of MetS. Metabolic alterations exert a significant pathological effect on myocardial structure and function, leading to HF [19]. A recent study by Alex et al. provides a very detailed analysis of HF in db/db mice, suggesting diastolic dysfunction with preserved ejection fraction, cardiomyocyte hypertrophy, interstitial/perivascular fibrosis, and microvascular rarefaction. Moreover, the severity of symptoms is sex-specific, with females exhibiting moderate hypertension, and males exhibiting decreased microvascular density [20].

Our results show a significantly higher body weight of db/db mice compared with that of age-matched controls, which is fairly consistent with previously published data [20,21]. Db/db mice gain weight rapidly and reach a plateau by about week 15. Body weight changes correlate with blood glucose levels. Interestingly, we observed a rapid elevation of blood glucose levels; however, after db/db mice reached week 15, their blood glucose levels dropped and did not significantly differ from those in lean controls. This result is similar to the findings of Alex et al. and Puff et al., who also observed a decrease in blood glucose levels; however, this drop was noticed after week 12, probably due to a compensatory mechanism through which an increase in beta cell mass contributes to enhanced insulin production [20,21].

Interestingly, cardiac hypertrophy (quantified by the heart weight-to-tibia length ratio) was not observed in db/db mice, with lower heart weight values in db/db mice compared with those in lean controls. Literature data differ in this respect, and results depend on the method used and animal age, gender, and other comorbidities. Choi et al. did not show any difference in heart mass between control and obese mice [22]; on the contrary, Wilson et al. observed a decrease in heart/body weight ratio in db/db mice and controls [23]. Finally, Wang et al. demonstrated an increase in heart weight/tibia length ratio in db/db mice [24]. These inconsistent results may be due to different methodology, although in conditions when body weight changes rapidly, cardiac hypertrophy is better quantified by relating heart weight to tibia length [25]. Of importance, two different processes simultaneously occur in the db/db mouse cardiac tissue: cardiomyocyte hypertrophy and elevated cardiomyocyte apoptosis. Therefore, a balance between these processes may, or may not, affect the heart weight, as it has been recently described by Papinska et al. [26].

Recently cardiac tissue macrophages have been reported to be involved in tissue repair regulation, angiogenesis, fibrosis, sensing of tissue edema and salt overload, and many other functions as well as their well-known phagocytic activity and immune cell modulation [27]. In pathological conditions, such as myocardial infarction, two populations of monocytes (Ly-6C^hi^ and Ly-6C^low^) become mobilized in the circulation. Ly-6C^hi^ monocytes invade cardiac tissue and differentiate into Ly-6C^hi^ macrophages, which are involved in dead cell phagocytosis and pro-inflammatory cytokine production. Furthermore, once acute inflammation resolves, infiltrating Ly-6C^hi^ monocytes become Ly-6C^low^ macrophages, which are associated with the regenerative process [28]. We used the common leukocyte marker CD45 together with standard macrophage markers CD11b, CD64, and Ly-6C to sort two populations of cardiac macrophages—pro-inflammatory CD45^+^CD11b^+^CD64^+^Ly6C^hi^ and pro-regenerative CD45^+^CD11b^+^CD64^+^Ly6C^low^ [29]. There have been no reports on the number of macrophage subsets in diabetic mouse cardiac tissue. Our results showed a lower number of the total macrophage population in db/db animals compared with that in healthy myocardia. In contrast, the ischemic tissue after myocardial infarction and the non-ischemic tissue in hypertrophic cardiomyopathy contain an elevated macrophage density [30,31]. Moreover, macrophage profiles and timing seem to play important roles in cardiac remodeling after myocardial infarction and in non-ischemic hypertrophy, by exhibiting either detrimental or beneficial effect depending on Ly6C^hi/low^ marker expression levels. Of note, the predominant population of macrophages in a healthy adult mouse heart consists of Ly-6C^low^ cells known for their vessel patrolling function, and with their number decreasing with age [32]. The differences between the total number of macrophages in the myocardia of db/db mice and the myocardia affected with ischemic or nonischemic heart disease might be due to the altered inflammatory profiles in ischemic and diabetic myocardia and, presumably, are related to animal age/disease stage and sex.

The Ly-6C^hi^ and Ly-6C^low^ macrophage subpopulations differ not only in surface marker expression but also in mRNA transcription profiles [33]. A novel finding of this study was identifying the miRNA transcription profile in Ly-6C^hi^ versus Ly-6C^low^ macrophages and in healthy versus db/db cardiac macrophages. We found 150 miRNAs that were expressed differently in control versus db/db mouse macrophages. Moreover, at least some of those miRNAs may be related to the pathogenesis of MetS (Table 1) and when expressed by macrophages or internalized via phagocytosis may be responsible for the modulation of macrophage phenotype (Table 2). Wang et al. demonstrated that activated macrophages release vesicles packed with miR-155. These vesicles can be taken up by cardiac fibroblasts, and the miR-155 molecules they contain subsequently downregulate fibroblast proliferation and promote the expression of fibroblast inflammatory response genes. Thus macrophage-derived miR-155 packed in exosomes seems to inhibit cardiac repair after myocardial infarction [34]. Zhang et al. described macrophage-released miR-150 that can be transferred into endothelial cells via exosomes and modify their metabolism. Thus, crosstalk between macrophages and endothelial cells could be an underlying mechanism of endothelial cell dysfunction and vascular injury in various conditions, including MetS [35]. Interestingly, the expression levels of selected miRNAs in cardiac tissue were not affected (Figure 5c). We compared the expression of miR-31, miR-23, and miR-27 isolated from cardiac macrophages with the expression of these miRNAs obtained from whole cardiac tissue. Whole-tissue miRNA analysis did not show any differences in these miRNA levels. This result may indirectly show that at least some of the analyzed miRNAs are synthetized by macrophages, but this requires further research.

PubMed search analysis revealed that at least 15 miRNAs differentially expressed in cardiac macrophages in db/db mice target multiple mRNAs for synthesis of proteins involved in inflammation (miR-21a, miR-26a, miR-27b, miR-29b, miR-30a, miR31, miR-126, miR-146, miR-223), fibrosis (miR-20a, miR-21a, miR-27b, miR-29b, miR-30a, miR-31, miR-146, miR-223), angiogenesis (miR-15a, miR-23a, miR-26a, miR-27b, miR-29b, miR-30a, miR31, miR-126, miR-146a, miR-148, miR-342), and lymphangiogenesis (miR-31), as summarized in Table 1. Moreover, the same miRNAs, when produced by macrophages or phagocytosed by these cells, may affect pro-inflammatory (miR-15a, miR-21a, miR-23a, miR-31, miR-92a-3p) or anti-inflammatory (miR-15a, miR-21a, miR-26a, miR-27b, miR-30a, miR-126a, miR-146a, miR-148b, miR-223, miR-342) phenotype modulation, phagocytosis intensity (miR29b), angiogenesis (miR-20a) or lipid uptake (miR-342). Importantly, all but miR-31 were downregulated in cardiac MetS Ly-6C^low^ macrophages compared to miRNA levels in Ly-6C^low^ macrophages of healthy animals. This effect was not observed in Ly-6C^hi^ populations which may indicate that “regenerative” subpopulation of macrophages is more affected by MetS condition than the “inflammatory” one. We are aware that multiple pathways are influenced by a combination of these miRNAs, each one of which might activate or inhibit cardiac fibrosis (as demonstrated in Figure 6a–d), microvessel involution (as shown in Figure 6e–g), and/or inflammatory cell profiles (Figure 6h). This paper presents novel data on miRNA expression profiles in isolated macrophages from db/db mouse myocardia. We discuss some of our results related to MetS effects on myocardial remodeling and compare them with those found in the literature. Still, there are some papers describing miRNA levels in plasma or in the heart tissue of individuals suffering from MetS or T2D, which might reflect microenvironmental influence on macrophage content. For example, some authors described downregulation of miR-126, miR-15a, miR-29b, and miR-223 in the plasma of diabetic patients, of patients with CAD, and of Lep^ob^ mice [36,37], whereas He et al. observed downregulation of miR-21 in the serum of patients with MetS and in circulating monocytes of type 1 diabetic individuals [38]. These observations of miRNA levels in sera are in line with our data regarding cardiac macrophages. miR-30 is considered the most abundant in the heart and is involved in ventricular remodeling by various mechanisms. miR-30 levels are downregulated in such cardiovascular conditions as hypertension, diabetic cardiomyopathy, and myocardial infarction. Reduction of miR-30 causes cardiac fibrosis (via a Snail-dependent pathologic pathway), promotes autophagy, and decreases angiogenesis and cardiomyocyte hypertrophy [39]. Among the miRNAs evaluated in our study, only miR-31 was significantly elevated in db/db mouse cardiac macrophages. miR-31 expression is stimulated by the vascular endothelial growth factor (VEGF) and directly downregulates tumor necrosis factor superfamily-15 (TNFSF15), which is a negative modulator of angiogenesis crucial for vascular homeostasis [40]. In MetS, when cardiac tissue undergoes remodeling, there is upregulation of serum VEGF [41,42]; however, soluble VEGF receptor levels also increase, which may block proangiogenic action of VEGF. An increased level of miR-31 in serum of patients with T2D and microvascular complications was observed by others [43]. Upregulation of this miRNA was observed after myocardial infarction, and its deleterious effect on cardiac function was described [44]. Of note, miR-31 also downregulates the expression of Prox-1, a major transcription factor responsible for lymphatic endothelial cell identity, and impairs venous sprouting and lymphangiogenesis in embryonic development [45].

MiR-126 has been thoroughly studied and reported to be involved in angiogenesis, endothelial cell (EC) proliferation, EC survival, and sustaining physiological functions of ECs. Downregulation of this miRNA causes endothelial cell apoptosis, and therefore microvessel involution, inhibits EC invasion and proliferation, thus impairing angiogenic activity [46].

miRNA expression levels and functions are very often tissue- or condition-specific. For example, we observed downregulation of miR-29b in db/db myocardium-derived macrophages. Nonetheless, other authors reported conflicting results concerning the expression of this miRNA. Van Rooij et al. also observed downregulation of miR-29b in cardiac tissue after myocardial infarction, which is associated with fibrosis [47]. Conversely, Sassi et al. described upregulation of miR-29b in cardiac myocytes in pressure overload-induced cardiac hypertrophy and fibrosis; they also reported miR-29b-induced dysregulation of non-canonical Wnt signaling pathway, which can regulate myocardial fibrosis [48]. Similar issue affects the results obtained for regulation of macrophage phenotype by miRNAs. For example, miR-15a is considered as pro-inflammatory due to their interaction with mRNA for TNFAIP3-interacting protein 2 (TNIP2) that represses inflammation [49], but on the other hand it may also inhibit the expression of JAK2, which switches macrophage phenotype towards anti-inflammatory [50]. Therefore, all results obtained with miRNAs, both in vivo and in vitro, are extremely tissue-specific and rely much on the experimental approach.

Over the last several years, miRNAs, especially those associated with cardiac macrophages, have gained considerable attention as potential therapeutic targets in cardiovascular diseases. Considering the multitude of macrophage genotypic subsets and their tissue-related plasticity, thorough research is required for further medical interventions targeted for specific tissues/organs and devoid of negative side-effects) [51,52,53]. Precise understanding of the role of miRNAs in regulating inflammatory or regenerative processes in the cardiac tissue may result in the development of therapeutic strategies in MetS-induced heart failure. Some authors report tools which enable efficient miRNA delivery and ensure miRNA stability within tissue [17], whereas others question their efficiency [54]. Circulating miRNA profiles can also be used as a diagnostic tool as they often correlate with the severity of cardiovascular events such as myocardial infarction [55]. In this paper we described for the first time the miRNA transcription profiles in two distinct macrophage populations in MetS-affected cardiac tissue. Results of our study might suggest that at least these few selected miRNAs are of macrophage origin. However, we cannot be certain whether the miRNAs detected in macrophage subpopulations are actually produced by macrophages or are released by other cells and subsequently phagocytized, as suggested by other authors [56,57]. Of note, some miRNAs within myocardial db/db mouse macrophages were down-regulated in comparison with the miRNA levels in control mouse macrophages. Therefore, downregulation of crucial remodeling processes by the absence or low levels of miRNAs might occur in cardiac tissue. Although our results are only preliminary, we believe that they may help elucidate macrophage function in MetS-related cardiac pathologies in the future.

## 4. Materials and Methods

### 4.1. Animals

This study was performed on BKS.Cg-Dock7<m>+/+Lepr<db>/J mice (db/db); the C57BL/6J strain was used as control. All animal experiments were approved by the First Local Bioethics Committee of the University of Warsaw, Poland and carried out in accordance with EU Directive 2010/63/EU for animal experiments. Nine-week-old male mice were purchased from Charles River (Italy) and kept under specific pathogen-free conditions, with unlimited access to LabDiet^®^ 5K52 (6% fat) chow (Charles River Laboratory, Sant’Angelo Lodigiano, Italy). After 1 week of adaptation, mouse blood glucose levels and body weight were measured every week. Blood samples were taken from mouse tails, and glucose levels were measured with a OneTouch Select Plus^®^ blood glucose meter (LifeScan, Milpitas, CA, USA). At the age of 21 weeks, the animals were sacrificed by CO_2_ asphyxiation, and their hearts were isolated for further analysis.

### 4.2. Assessment of Macrophage and Microvascular Density in a Confocal Microscope

Frozen hearts were cut serially into 10-μm sections; subsequently, the sections were fixed in 4% paraformaldehyde; washed with PBS; incubated with 1% BSA, 0.1% TritonX-100, and 0.1 M glycine in PBS for 30 min; and blocked with 10% donkey serum (Jackson ImmunoResearch Laboratories, West Grove, PA, USA). For macrophage density assessment the sections were incubated for 1 h with primary antibodies against the CD68 molecule (Abcam, Cambridge, UK, cat. no ab125212, final concentration 1:100) diluted in PBS containing 5% donkey serum, followed by two washes in PBS. Then, the slides were incubated with Cy™3-conjugated donkey anti-rabbit IgG, (Jackson ImmunoResearch, Laboratories West Grove, PA, U.S. cat. no 711-165-152, final concentration 1:800) and Wheat Germ Agglutinin (WGA), Alexa Fluor 488 Conjugate (Molecular Probes, Eugene, Oregon, USA, cat. no W11261, final concentration 1:1800) diluted in PBS/1% BSA for 1 h. In order to assess microvascular density, the sections were incubated for 1 h with primary antibodies against CD31 (BD Biosciences, San Jose, CA, USA, cat. no 550274, final concentration 1:100) and Lyve-1 molecules (Angiobio, San Diego, CA, USA, cat no. 11-034, final concentration 1:100) diluted in PBS with 5% donkey serum, followed by two washes in PBS. Subsequently, the slides were incubated with Cy™3-conjugated donkey anti-rabbit IgG and donkey anti-rat AlexaFluor™647 (Jackson ImmunoResearch Laboratories West Grove, PA, U.S., cat. no 711-165-152, final concentration 1:800 and cat. no 712-605-153, final concentration 1:500, respectively). Cell nuclei were counterstained with DAPI (Thermo Fisher Scientific, Waltham, MA, USA). Sections mounted in Fluorescence Mounting Medium (Dako, Glostrup, Denmark) were viewed under a Leica confocal microscope (Leica, Wetzlar, Germany). Most representative figures were chosen from three heart samples (i.e., immunostained scans of tissue sections) from each group of animals. Macrophage locations and numbers were assessed by evaluating the distribution of CD68-positive cells per myocardial tissue sections area in various locations: the left ventricular wall, right ventricular wall, and septum. Microvascular rarefaction was assessed by evaluating the number of CD31-positive/Lyve-1 negative cells in the left ventricle. 10 regions of interest were randomly selected, and the images were taken under a 20× objective. To avoid inter-counter variation, all countings were performed by the same operator. Data are expressed as the mean number of cells per area of 1 mm^2^.

### 4.3. Cardiac Macrophage Isolation by Flow Cytometry Sorting

Hearts were collected from 21-week-old db/db and control mice, cut in half, and rinsed in PBS. Next, the hearts were cut into pieces and digested with 0.5 mg/mL collagenase type II (Sigma-Aldrich, St. Louis, MO, USA) on a magnetic stirrer at 37 °C for 45 min. To obtain single cell suspensions, the digested tissue was pipetted and filtered through 40-µM nylon filters (Falcon, Corning, New York, NY, USA). The cells were washed twice and suspended in a staining buffer (1% BSA in PBS). First, the cells were incubated with Fixable Viability Dye (eBioscience, San Diego, CA, USA, cat no 65-0865-14, Thermo Fisher Scientific, Waltham, MA, USA). Then Fc receptors (CD16/CD32) were blocked with Fc Block (cat no, 553141, BD Biosciences, San Jose, CA, USA). The antibodies were as listed: CD45 (clone 30-F11, cat. no 563891, BD Biosciences, San Jose, CA, USA), CD11b (clone M1/70, cat. no 562605, BD Biosciences, San Jose, CA, USA), CD64 (clone X54-5/7.1, cat. no 558539, BD Biosciences, San Jose, CA, USA), Ly6C (clone AL-21, cat. no 560592, BD Biosciences, San Jose, CA, USA). Stained cells were washed, suspended in PBS, sorted with FACSAria I, and analyzed with BD FACSDiva software (Becton-Dickinson, Franklin Lakes, NJ, USA). Cardiac macrophages were identified as CD45^+^CD11b^+^CD64^+^ cells and sorted into two subpopulations based on Ly6C expression (Ly6C^+/hi^ and Ly6C^−/low^). The sorting strategy is shown in Figure 3a.

### 4.4. Inflammatory Cell Profile of Cardiac Tissue Cell Suspension

The cell suspension from cardiac tissue was prepared as described above. Cells were incubated with Fixable Viability Dye (eBioscience, San Diego, CA, USA, cat. no 65-0865-14, Thermo Fisher Scientific, Waltham, MA, USA). The cardiac inflammatory cell profile was evaluated based on the following markers: CD45 (clone 30-F11, cat. no 563891, BD Biosciences), CD19 (clone 1D3/CD19, no cat. 152404, BioLegend, San Diego, CA, USA), CD3 (clone 17A2, cat. no 100217, BioLegend), CD8a (clone 53-6.7, cat. no 100722, Biolegend), CD4 (clone RM4-5, cat. no 100530, BioLegend), CD25 (clone 3C7, cat. no 101904, BioLegend), and Ly6G (clone 1A8, cat. no 127628, BioLegend). The stained cells were washed, suspended in PBS, and analyzed using flow cytometry (FACSCanto II, Becton-Dickinson).

### 4.5. RNA Isolation, Total miRNA Library Preparation, and miRNA Sequencing

After cells were transferred to a lysis solution, RNA was isolated with a mirVana™ miRNA Isolation Kit (Thermo Fisher Scientific, Waltham, MA, USA) and purified with DNAse I. The initial RNA concentrations were measured with NanoDrop One/OneC Microvolume UV Spectrophotometer (Thermo Fisher Scientific, Waltham, MA, USA). At least 60 ng of each RNA was used for further experiments. Total miRNA libraries were prepared with an miRNA Library kit and miRNA NGS 12 Index IL kit (Qiagen, Venlo, The Netherlands) according to the manufacturer’s instruction. Quality assessment of the miRNA libraries was performed with the Agilent 2100 Bioanalyzer (Agilent, Santa Clara, CA, USA) and High Sensitivity DNA chips (Agilent, Santa Clara, CA, USA). Final library concentrations were measured with a Qubit dsDNA High Sensitivity Kit (Thermo Fisher Scientific, Waltham, MA, USA) and sequenced on the MiSeqDx Instrument (Illumina, San Diego, CA, USA).

### 4.6. miRNA-Seq Data Analysis

The results were analyzed with the Data Analysis Center available on the Qiagen website www.qiagen.com (accessed on 22 February 2021). The results were normalized with the “Trimmed Mean of M” method and heat maps were prepared based on the geNorm method. Fold-Change represents the normalized miRNA expression of each Test Sample divided by the normalized miRNA expression of the Control Sample. The results of detected miRNAs were subsequently sorted according to their significance. Out of 1400 miRNAs, about 150 miRNAs, whose expression differed significantly between control and db/db mouse macrophages, were selected for further manual search in the PubMed database in order to verify their associations with MetS.

### 4.7. RT-PCR Analysis of Cardiac miRNAs

Thirty-nanogram pieces of cardiac tissue were transferred to lysis solution and homogenized. RNA was isolated with a mirVana™ miRNA Isolation Kit, and the initial concentrations and quality of RNA were measured with NanoDrop (Thermo Fisher Scientific, Waltham, MA, USA). Reverse Transcription was performed with a TaqMan MicroRNA Reverse Transcription Kit according to the manufacturer’s protocol (Applied Biosystems, ThermoFisher Scientific, Waltham, MA, USA) with RT primers: snoRNA234 (RT:001234), snoRNA202 (RT:001232), hsa-miR-23a (RT:000399), has-miR-27b (RT:000409), and mmu-miR-31 (RT: 000185). cDNA was stored at −20° C. Gene expression was measured with the relative quantitation (RQ) using a comparative C_T_ assay [58] Cardiac tissue from control mice was used as a calibrator. Real-Time PCR was performed in Abi Prism 7500 Applied Biosystems, ThermoFisher Scientific, Waltham, MA, USA) in 96-well optical plates. Each sample was run in triplicates and supplied with endogenous controls snoRNA234 (TM:001234) and snoRNA202 (TM:001232). For miRNA quantification TaqMan Expression Assays were used: hsa-miR-23a (TM:000399), has-miR-27b (TM:000409), mmu-miR-31 (TM: 000185). All probes were stained with FAM (all from, Applied Biosystems, ThermoFisher Scientific, Waltham, MA, USA). Reactions were run in a 20-μL volume with TaqMan Universal Master Mix (Applied Biosystems, ThermoFisher Scientific, Waltham, MA, USA), appropriate primer set, MGB probe, and 5 ng of cDNA template. Universal thermal conditions, i.e., 10 min at 95 °C, 40 cycles of 15 s at 95 °C, and 1 min at 60 °C, were used. Data analysis was done with sequence detection software version 1.2 (Applied Biosystems, ThermoFisher Scientific, Waltham, MA, USA).

### 4.8. Picrosirius Red Staining for Collagen Deposits

Hearts from age-matched control and db/db mice were fixed in buffered 4% paraformaldehyde (pH 7.2), rinsed in water, and processed for paraffin blocks. Paraffin sections were deparaffinized and routinely stained with hematoxylin-eosin and with Picrosirius red (for collagen deposits) with modification according to Puchler and/or Junqueira [59,60]. Briefly, sections were stained with Weigert’s hematoxylin, followed by immersion in phosphomolybdic acid, and subsequently stained in 2% Picrosirius red solution for 60 min. After clearing in 95% ethanol, the sections were mounted in a histologic mounting medium.

**Table 1:** Selected miRNAs that are potentially involved in MetS pathogenesis, with some of their confirmed targets.

miRNA	Function	Target Gene	References
miR-15a-5p	Angiogenesis, fibrosis	*Tie-2*, fibrosis via VEGF and EMT; and TGF-β1/Smad2	[61,62,63]
miR-20a-5p	fibrosis	*ALK-5, TGFβR2, SARA, CD36*	[64,65]
miR-21a-5p	fibrosis, inflammation	*PPARα, SMAD7, PTEN*	[66,67,68]
miR-23a-3p	angiogenesis	*SEMA6A, Sprouty2*	[69,70]
miR-26a-5p	inflammation, angiogenesis	*PTEN, VEGF-A, PI3K/AKT*	[71,72]
miR-27b-3p	fibrosis, inflammation, angiogenesis	*ALK5, IL-1, IL-6, TNFα, MCP1, SEMA6A, Sprouty2*	[69,73,74,75,76,77]
miR-29b-3p	fibrosis, extracellular matrix deposition, angiogenesis	*Wnt, elastin, collagen, fibronectin, IGF-1*	[47,48,78,79]
miR-30a-5p	fibrosis, inflammation, angiogenesis	*Angiopoietin-2, VCAM-1, CTGF, Beclin1, Dll4, Snail1, Wnt*	[80,81,82,83,84,85,86]
miR-31-5p	lymphangiogenesis, fibrosis, angiogenesis, inflammation	*TNFSF15, PKCε, Prox1, FOXC2, E-selectin*	[40,45,87,88]
miR-92a-3p	angiogenesis	*KLF2, KLF4, TF*	[89,90]
miR-126a-3p	angiogenesis, inflammation	*VCAM-1, Spred-1, PI3KR2, VEGF-A*	[91,92,93,94,95]
miR-146a-5p	inflammation, fibrosis, angiogenesis	*NFκ* *B, TRAF6, IRAK, MYD88, SMAD4, MAPK*	[96,97,98]
miR-148b-3p	angiogenesis, fibrosis, EMT	*FGF-2, DNMT, PTEN, Wnt-β-catenin*	[99,100,101]
miR-223-3p	inflammation, fibrosis	*ICAM-1, RASA1, FBXW7*	[102,103,104]
miR-342-3p	angiogenesis	*FGF11*	[105]

**Table 2:** Selected miRNAs that are potentially involved in MetS pathogenesis due to their involvement in macrophage phenotype regulation. Please note that contradictory results are due to different experimental approach and model used.

miRNA	Target Gene	Effect on Macrophage and on Macrophage Phenotype Alteration	References
miR-15a-5p	*TNIP2* *JAK2*	Pro-inflammatory in vitro and in mouse model of sepsis Anti-inflammatory in in vitro model of allergic rhinitis	[49,50]
miR-20a-5p	*HIF-2α*	Anti-angiogenic in tumor associated macrophages (TAMs)	[106]
miR-21a-5p	*PDCD4* *IL-10*	Anti-inflammatory in *Brucella* infected bone marrow derived macrophages Suppresses M-2 macrophage polarization in primary bone-marrow derived macrophages during particle-induced osteolysis	[107,108]
miR-23a-3p	*TNFAIP3*	Tumor associated macrophages (TAMs) switching towards M1 phenotype	[109]
miR-26a-5p	*CTGF*	Reduces pro-inflammatory factor TNF-α, IL-6, IL-1β expression of LPS-induced mouse alveolar macrophages	[110]
miR-27b-3p	*MIP-1β*	Anti-inflammatory effect in bone-marrow derived macrophages in vitro	[111]
miR-29b-3p	*DNMTs* (confirmed indirectly)	Inhibits phagocytic function in alveolar macrophages in syngeneic murine model of bone marrow transplantation	[112]
miR-30a-5p	*SOCS3*	Anti-inflammatory in LPS stimulated RAW 264.7 macrophages	[113]
miR-31-5p	*Cab39*	Pro-inflammatory in LPS-induced alveolar macrophages in vitro	[114]
miR-92a-3p	*PTEN* *KLF4*	Pro-inflammatory in LPS-induced acute lung injury mouse model Pro-inflammatory in monocyte-derived macrophages under atheroprone microenvironment in vitro	[115,116]
miR-126a-3p	*ATF3, ATP1B1,* *ATP9A* and *RAI14*	Reduction of pro-inflammatory cytokine/ chemokine secretion by primary human macrophages and increase in their phagocytic activity	[117]
miR-146a-5p	*Notch1*	M2 phenotype polarization of microglia following brain stroke in vivo and in vitro	[118]
miR-148b-3p	*Nox2*	Anti-inflammatory and lowering ROS production in macrophages in myocardial infarction mouse model	[119]
miR-223-3p	*Nlrp3* *Pknox1* *Rasa1* and *Nfat5*	Anti-inflammatory in acute and chronic hepatic injury, and in vitro studies M2 phenotype polarization in bone marrow–derived macrophages stimulated with LPS and in adipose tissue of mice on a high-fat diet M2 phenotype polarization in adipose-tissue macrophages isolated from mice on a high-fat diet	[120,121,122]
miR-342-3p	*Chi3l1* (not confirmed)	Suppresses inflammation and lipid uptake in THP-1 cells	[123]

### 4.9. Statistical Analysis

Blood glucose, body weight, macrophage count, microvascular rarefaction, and miRNAs expression level statistics were calculated with SAS 9.4 software, and graphs were prepared with SAS ODS Graphics Editor 9.43. Sample distribution was measured with the Kruskal–Wallis test, and the null hypothesis was tested with Wilcoxon signed-rank test. Normal distribution was evaluated with Shapiro-Wilk test. For the “Trimmed Mean of M” normalization method of NGS results the *p*-values were calculated with Bioconductor software.

## Data Availability

The datasets generated during and/or analysed during the current study are available from the corresponding author.

## Supplementary Materials

The following are available online at https://www.mdpi.com/1422-0067/22/4/2197/s1. Table S1. Analysis of miRNA expression in cardiac macrophage populations. Numbers represent fold change that is the normalized miRNA expression in each sample (db/db mouse CD45^+^CD63^+^CD11b^+^Ly6C^+/hi^ and CD45^+^CD63^+^CD11b^+^Ly6C^−/low^ cardiac macrophages) divided the normalized miRNA expression in the control sample (control mouse cardiac CD45^+^CD63^+^CD11b^+^Ly6C^+/hi^ macrophages).
